# The Role of TGF**β** Signaling in Squamous Cell Cancer: Lessons from Mouse Models

**DOI:** 10.1155/2012/249063

**Published:** 2012-12-26

**Authors:** Adam B. Glick

**Affiliations:** Department of Veterinary and Biomedical Sciences, The Pennsylvania State University, University Park, PA 16802, USA

## Abstract

TGF**β**1 is a member of a large growth factor family including activins/inhibins and bone morphogenic proteins (BMPs) that have a potent growth regulatory and immunomodulatory functions in normal skin homeostasis, regulation of epidermal stem cells, extracellular matrix production, angiogenesis, and inflammation. TGF**β** signaling is tightly regulated in normal tissues and becomes deregulated during cancer development in cutaneous SCC and many other solid tumors. Because of these diverse biological processes regulated by TGF**β**1, this cytokine and its signaling pathway appear to function at multiple points during carcinogenesis with distinct effects. The mouse skin carcinogenesis model has been a useful tool to dissect the function of this pathway in cancer pathogenesis, with transgenic and null mice as well as small molecule inhibitors to alter the function of the TGF**β**1 pathway and assess the effects on cancer development. This paper will review data on changes in TGF**β**1 signaling in human SCC primarily HNSCC and cutaneous SCC and different mouse models that have been generated to investigate the relevance of these changes to cancer. A better understanding of the mechanisms underlying the duality of TGF**β**1 action in carcinogenesis will inform potential use of this signaling pathway for targeted therapies.

## 1. Pathogenesis of Squamous Cell Carcinoma 

Nonmelanoma skin cancer including both basal cell carcinoma and squamous cell carcinoma is the most frequent cancer among Caucasian populations, with incidence rates matching all other cancers combined in these groups [1]. Although exposure to ultraviolet radiation from the sun is the major risk factor for cutaneous squamous cell carcinoma (SCC), other risk factors also include chronic inflammation, and wounding, as well as exposure to arsenic, tobacco, and coal tar products [2]. The multistage mouse skin carcinogenesis model has been instrumental in defining the basic biology of SCC development in the skin and other epithelia. Mice are treated once with a carcinogen such as the polycyclic aromatic hydrocarbon dimethylbenz[a]-anthracene (DMBA) followed by 20 weekly applications of a nonmutagenic agent such as 12-*O*-tetradecanoylphorbol-13-acetate (TPA) that provides a microenvironment and proliferative stimulus that favors clonal outgrowth of initiated keratinocytes. Benign papillomas representing clonal outgrowths of keratinocytes with initiating mutations in the *Hras1 *arise within 10–15 weeks. These are largely exophytic hyperplastic and hyperkeratotic lesions that retain the stratified organization of the normal epidermis and retain expression of normal differentiation markers such as keratin 1 and keratin 10. Many of these lesions are also promoter dependent, and if the stimulus is removed, tumor regression occurs. In the most benign lesions proliferation is confined to the basal layer as in the normal epidermis. Tumor progression in this model is associated with focal loss of keratin 1 and 10, expression of keratin 13, a keratin not normally expressed in the epidermis, expansion of the proliferative compartment, and changes in integrin expression. At the genetic level, tumor progression is associated with trisomy of chromosomes 6 and 7, loss of heterozygosity at the *Hras1* locus, followed by amplification of the mutated ras gene, and increasing aneuploidy [3]. Many additional genetic changes and signaling pathways that have been identified in this model are important for tumor promotion and progression and applicable to development of human SCC [4, 5]. In the typical 2-stage model using inbred strains such as SENCAR A which are highly sensitive to tumor promoters, most papillomas do not convert to SCC. A number of studies have documented the existence of subpopulations of papillomas with differing potential for malignant progression, and at early time points, this is reflected in distinct patterns of gene expression [6, 7].

## 2. TGF***β***1 Signaling Pathway Overview

**Figure 1:**
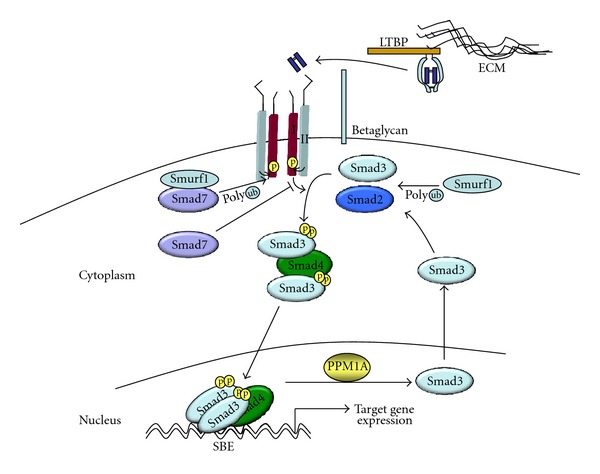
Schematic of TGF*β*1 signaling pathway and its regulation. TGF*β*1 is secreted and sequestered in the extracellular matrix as a biologically inactive complex composed of the TGF*β*1 peptide linked to the latency-associated peptide (LAP) and a member of the latent TGF*β*-binding protein (LTBP) family. Activation of latent TGF*β*1 allows binding of active peptide dimer to T*β*RII and formation of a heterotetrameric receptor complex Between T*β*RI and T*β*RII. Coreceptors such as betaglycan act to enhance TGF*β* binding to its receptors. T*β*RII, phosphorylates the cytoplasmic domain of T*β*RI and activates its serine-threonine kinase activity towards the R-Smads, Smad2, or Smad3, Phosphorylation of an R-Smad for allows complex formation with Smad4 and translocation to the nucleus, where binding to SBE target sites in gene promoters activates transcription with many other cofactors. Dephosphorylation of R-Smads by Smad phosphatases such as PPM1A attenuate signaling and cause Smads to recycle to the cytoplasm. Smad7 can block type I receptor phosphorylation of R-Smads and in conjunction with E3 ubiquitin ligases such as Smurf1 cause polyubiquitination and degradation of T*β*RI. Smurf1 and similar proteins have also been implicated in degradation of R-Smads.

From its initial identification as a major negative regulatory pathway for epithelial cell proliferation, Transforming growth factor-beta (TGF*β*1) and its signaling pathway has been identified as a critical regulator of cancer development and progression in humans and in many experimental cancer models in mice [8, 9]. The cell surface receptor for TGF*β*1 is a complex of TGF*β*1 type I and type II transmembrane receptors (T*β*RI and T*β*RII), both of which are serine threonine kinases. Binding of TGF*β*1 to T*β*RII recruits T*β*RI into a heterotetrameric complex resulting in phosphorylation and activation of the cytoplasmic domain of T*β*RI by T*β*RII kinase (Figure 1). This activates the kinase activity of the T*β*RI towards its substrates the R-(receptor activated) Smads which for TGF*β*1 and activin are Smad2 and Smad3. Smad1, 5, and 8 are R-Smads activated by BMP and its specific transmembrane receptors. Once phosphorylated, Smad2 or Smad3 form a complex with the co-Smad, Smad4, and translocate to the nucleus to regulate TGF*β* responsive genes, through either specific Smad-binding elements, other suppressive elements or through interaction with other transcription factors [10, 11]. TGF*β*s can also activate members of the mitogen-activated protein (MAP) kinase signalling molecules, including JNK, p38, ERKs, and the PI3 K/AKT pathway [9].

There are a number of mechanisms for downregulating or inhibiting TGF*β* signaling, including phosphatases which dephosphorylate Smad2 and 3 and attenuate signal strength [12, 13] inhibitory or I-Smads, Smad6, and Smad7 which block type I receptor phosphorylation of R-Smads [14, 15] and also recruit Smad ubiquitin regulatory factor 1 (Smurf1) and Smurf2 ubiquitin ligases to cause degradation of the type I receptor and Smads [16]. Other ubiquitin ligases such as the HECT (homologous to the E6-accessory protein C-terminus)-type E3 ubiquitin ligases are also important in regulating Smad levels [17]. Additional cell surface coreceptors, predominantly betaglycan, and endoglin modulate TGF*β*1 family members binding to their signaling receptor [18, 19]. There are three TGF*β*'s: TGF*β*1, *β*2, and *β*3, with similar but not identical receptor affinities and biological activity, and distinct patterns of expression [20]. All bioactive TGF*β*'s are 25 Kd disulfide-linked homodimers generated from the C-terminal 112 amino acids of the primary translation product (390 amino acids for TGF*β*1) [21]. Production of bioactive TGF*β* is also a complex process. TGF-*β*1 is secreted as a biologically inactive molecule called the small latent complex (SLC) that is unable to bind to its receptor [22]. The SLC consists of the active cytokine noncovalently linked to its propeptide called the latency-associated peptide (LAP) [22]. Additional proteins known as latent TGF*β*-binding proteins LTBP-1, 3, and 4 form disulfide bonds with the LAP to generate the large latent complex [23]. The LTBPs are structurally similar and part of the fibrillin protein family, an extracellular matrix protein. TGF*β*s are secreted as a complex termed the large latent complex (LLC) in which the LTBP is covalently bound to the TGF*β* propeptide, and on secretion, the (LLC) may be covalently linked to the extracellular matrix (ECM) [24]. Several mechanisms for the activation of latent TGF*β* complexes are known, and a diverse group of activators, including proteases, thrombospondin-1, the integrin *α*_v_ *β*_6_, reactive oxygen species (ROS), and low pH can activate TGF*β* [23–25].

## 3. Alterations in TGF***β***1 Pathway in Human SCC

 A number of immunohistochemical and mutational analysis studies have been done in human SCC to determine what changes in the TGF*β* signaling pathway are associated with tumor development. A number of studies in human head and neck SCC (HNSCC), cutaneous, and cervical SCC have been done by IHC with both increase and decrease relative to adjacent normal tissue reported. In studies with largest sample siz,e the results support a decrease in TGF*β*1 expression in HNSCC and cervical SCC [26–29], while other studies have shown an increase in TGF*β*1 expression in human cutaneous SCC [30]. It is not clear if the tumors with elevated ligand expression represent a distinct subset of tumors, but we and others have linked decreased or loss of TGF*β*1 expression with increased risk for malignant progression in the 2-stage skin carcinogenesis model [31, 32]. A number of different mutations in both the type I and type II receptors with distinct biological properties have been identified in HNSCC, but these are present at low frequency (up to 10%) in human SCC. In contrast, downregulation of expression of either receptor is much more frequently observed in up to 60% of tumor samples (see Xie and Riess, 2011, for comprehensive review) [33]. Only a handful of mutations in the Smad2 or Smad4 genes have been identified in human SCC, and none for Smad3. However, loss of heterozygosity (LOH) has been observed in Smad4; LOH occurs in 30–50% of HNSCC and esophageal SCC tumors and cell lines [34–37]. In a sample of 36 HNSCC Smad4 mRNA levels were reduced by about 50% compared to normal control mucosa in 86% of tumors, and Smad4 protein was reduced or not detected similarly [35]. Similarly in a sample of 85 human skin SCC, Smad4 and Smad2 proteins were each absent in 70-% of the tumors relative to normal skin, with Smad2 loss observed in 100% of poorly differentiated tumors. A similar reduction in Smad2 and Smad4 mRNA levels in poorly differentiated tumors was also observed [38]. In two large tissue array studies of HNSCC (170 and 340 samples), 18.5% had no detectable expression of phospho-Smad2, 40% had no detectable phospho-Smad3 (indicating likely downregulation of the pathway), and 12% did not express Smad4 [39, 40]. Among 198 patients with survival information, those with pSmad2/pSmad3 negative tumors had a better overall survival rate compared to those with pSmad2-positive SCC [40]. It is not clear whether the wide variance in percentage of skin or HNSCC exhibiting loss of Smad immunostaining represents differences in patient population or methodology. Nevertheless, loss of components of the TGF*β*1-signaling pathway represent a significant component of HNSCC and cutaneous SCC pathogenesis. 

## 4. Mouse Models of Altered TGF***β***1 Signaling in Skin Cancer

### 4.1. TGF*β* Receptors

 Mice expressing a dominant negative *Tgfbr-2* (delta-T*β*RII) transgene in the basal and suprabasal epidermis exhibited a hyperkeratotic and thickened skin at birth, with increased basal and suprabasal proliferation and altered differentiation [41]. Primary keratinocytes from these mice were resistant to TGF*β*1-induced growth inhibition as expected [41]. In a 2-stage chemical carcinogenesis experiment with the delta-T*β*RII mice, benign papillomas appeared 2 weeks earlier than in control nontransgenic mice, and there was a 2-fold increase in tumors from 4 per mouse to 8 per mouse in the delta-T*β*RII mice [42]. While many papillomas that arise in the 2-stage model are promoter dependent, and regress when promotion is stopped, papillomas that formed in delta-T*β*RII mice did not regress when TPA promotion was stopped but progressed rapidly to squamous cell carcinoma [42] (Table 1). This suggests that suppression of TGF*β*1 signaling converts benign tumors from promoter dependent to promoter-independent lesions, a characteristic of tumors at high risk for malignant conversion. Surprisingly, TPA treatment alone induced papilloma formation suggesting that inhibition of TGF*β*1 signaling in some cells could act as an initiating event. Tumors from the delta-T*β*RII mice exhibited altered cell cycle regulation and reduced expression of TGF*β*1 regulated cyclin-dependent kinase inhibitors such as p15^ink4b^, p21^waf1^ and p27, but no evidence for chromosome instability [43]. Additionally tumors that formed in mice with blocked TGF*β*1 signaling had increased neovascularization and changes in expression of positive regulators of angiogenesis including vascular endothelial growth factor (VEGF) and TGF*β*1 and reduced expression of the angiogenesis inhibitor thrombospondin-1 [42]. However, it is not clear if these are direct effects of inactivation of TGF*β* signaling or simply reflective changes of a more progressed tumor phenotype.

 Using a complimentary approach several groups have generated tissue-specific conditional knockouts of the type 2 and type 1 receptor. Deletion of *Tgfbr2* in the epidermis and oropharyngeal epithelium with an inducible Keratin 5 (K5)-Cre by itself only caused slight epithelial hyperplasia after one year. However, when crossed onto mice expressing a K-Ras transgene or when *Tgfbr2* mice were initiated with DMBA, the development of SCC was greatly accelerated and some SCC became metastatic [53]. Similar to results with the DN-T*β*RII there was increased expression of TGF*β*1 in the head and neck tumors that developed which correlated with increased inflammation and angiogenesis [53]. Deletion of *Tgfbr2 *with a Keratin 14 (K14)-Cre transgene also had only mild effects on the epidermis, with increased epidermal proliferation balanced by increased apoptosis [54]. However, skin grafts of Ha-Ras^V12^ retrovirus transduced *Tgfbr2* null keratinocytes rapidly developed into large, aggressive tumors. Thus, loss of TGF*β* signaling reset epidermal homeostasis but did not by itself cause significant precancerous changes in the epidermis, but facilitates rapid malignant progression in the presence of oncogenic Ras. In contrast invasive SCC developed spontaneously in the anogenital epithelium, which also expresses K14, and this is likely due to the elevated basal proliferation and turnover in this tissue [54]. 

 Similar observations were made using K14-CreER mice to drive an inducible conditional deletion of the *Tgfbr1* gene, although the focus in these studies was epithelia of the oral cavity [51]. Again in the absence of initiating mutations, deletion of *Tgfbr1 *did not result in tumor formation, but with DMBA treatment HNSCC developed in approximately half of the mice, preceded by enhanced proliferation and decreased apoptosis in basal epithelial cells and activation of the PI3-kinase/AKT pathway [51]. In a recent followup study from this group, conditional deletion of both *Tgfbr1* and the tumor suppressor phosphatase and tensin homologue (PTEN) which inhibits the PI3-kinase/AKT pathway leads to rapid development of SCC with near complete penetrance. These tumors exhibit expansion of the putative cancer stem cell compartment, escape from senescence and an immunosuppressive inflammatory tumor microenvironment [52]. Taken together these results clearly show the tumor suppressor function of both type I and type II TGF*β* receptors, although the inactivation of this signaling pathway by itself does not appear to be enough to cause tumor formation. However, it is not clear why overexpression of the truncated dominant negative type II receptor has such profound effects by itself on epidermal homeostasis while deletion of either type I or type II has relatively mild effects. One possibility is that the truncated type II receptor is able to interact with and inhibit function of other type I receptors for members of the TGF*β* superfamily such as activin receptors, and this exaggerates the effect on epidermal hyperproliferation. Although speculative, interactions between TGF*β* and activin receptors have been described in endothelial cells [60], and epidermal-specific deletion of activin receptor type 1B causes epidermal hyperproliferation along with significant hair cycle defects [61]. Although by itself inactivation/loss of either TGF*β* receptor does not cause tumor formation, cooperation with either a RAS oncogene or activation of the PI3-kinase/AKT pathway through PTEN loss generates SCC in squamous epithelia. 

#### 4.1.1. Pharmacological Inactivation of TGF*β* Receptors

 A number of small molecule inhibitors of T*β*RI and related serine threonine kinases have been developed [62, 63] and been shown in a number of different cancer models to block TGF*β* responses in tumor cell lines and in cells in the tumor stroma [64, 65]. Two studies have been published using ALK5 inhibitors in the mouse skin carcinogenesis model. In the first, FVB/*n* mice were initiated with DMBA, and the ALK5 inhibitor SB431542 was applied topically during tumor promotion. Mice that were treated with TPA and SB431542 developed significantly fewer papillomas than TPA alone, but those tumors that did form had a higher frequency of conversion to SCC. SB431542 treatment blocked TPA-induced Smad2 phosphorylation in keratinocytes and dermal cells, and TPA-induced skin inflammation, suggesting that the induction of TGF*β*1 by TPA [66] and subsequent activation of signaling in keratinocytes and stromal cells is critical for tumor outgrowth, possibly through effects of TGF*β*1 on inflammatory gene expression [49]. Early papillomas that did form under conditions of inhibited TGF*β* signaling, however, had elevated intratumor inflammatory infiltrates and reduced expression of squamous differentiation, markers, similar to SCC. A subsequent *in vitro* study also provided evidence that pharmacologic inhibition of ALK5 with SB431542 induced terminal differentiation in primary mouse keratinocytes expressing an inducible oncogenic human H-*RAS* ^V12G^ transgene [67], and this could be an additional mechanism for suppression of papilloma formation. In a second chemical carcinogenesis study, mice were placed on systemic LY2109761, a potent inhibitor of both T*β*RI and T*β*RII, during tumor promotion. While in this study, the effect was seen on tumor incidence or latency, the SCC that formed under conditions of sustained type I/type II kinase inhibition had elevated levels of pSmad2 and appeared resistant to the drug and expressed markers of a more aggressive and invasive phenotype [50]. While it is not clear how topical versus systemic inhibition of TGF*β* signaling may differentially affect tumor formation, taken together these data suggest that subpopulations of initiated keratinocytes may respond differently to inhibition of TGF*β* signaling either within themselves or the tissue microenvironment. One population appears to require TGF*β* signaling for clonal expansion in response to TPA, while in the other inhibition of TGF*β*, it signaling appears to promote outgrowth and more rapid progression, possibly selecting for premalignant cells with pathway activation via a distinct mechanism.

### 4.2. Smads 

#### 4.2.1. Smad2

In a 2-stage chemical carcinogenesis study, *Smad2*+/− mice had accelerated skin tumor formation that was characterized by moderately differentiated SCC with local invasion [68]. Mice with a keratinocyte-specific *Smad2* deletion exhibited accelerated formation and malignant progression of chemically induced skin tumors compared with WT mice, and the Smad2−/− tumors were poorly differentiated and exhibited epithelial to mesenchymal transition (EMT) characterized by reduced E-cadherin expression [38]. In addition, these tumors were angiogenic and this was associated with epithelial overexpression of HGF and endothelial activation of the HGF receptor c-Met [55]. Both increased Snail and HGF expression in *Smad2*−/− tumors was directly linked to a switch from Smad2 repressive activity to increased binding of Smad4 to transcriptional coactivators at the Snail and HGF promoters [38, 55]. This study also provided evidence for a correlation of Snail and HGF expression in human SCC, where Smad2 expression was lost compared to Smad2 positive tumors. These studies contrast significantly with an earlier analysis of the role of Smad2 in conversion of murine squamous cell carcinoma to spindle cell carcinoma cell phenotype. Spindle cell carcinoma are a highly undifferentiated and invasive tumor type in the epidermis thought to result in part from an EMT of SCC cells, dependent on TGF*β*1 signaling [69, 70]. Overexpression of Smad2 in SCC cells in the context of elevated H-Ras causes EMT to a spindle cell phenotype and increases invasiveness and metastasis [71]. Although the conflict may arise from the analysis of Smad2 function in the context of the intact epidermis versus cell lines it is also possible that long-term loss of Smad2 in the epidermis causes compensatory mechanisms that generate the same phenotype as Smad2 overexpression. Nevertheless, loss rather than overexpression phenocopies human skin cancer [38]. However, it remains to be determined how the mouse model fits with observed increased survival of patients with pSmad2/pSmad3 negative HNSCC relative to to those with pSmad2-positive SCC [40]. Deletion of Smad2 in papillomas or SCC or conditional overexpression of Smad2 would help resolve these issues. 

#### 4.2.2. Smad3

 In two chemical carcinogenesis studies using Smad3+/− and Smad3−/− mice, it was found that in contrast to Smad2 deletion, Smad3+/− mice developed fewer tumors compared to wild-type controls [68]; *Smad3*−/− mice also developed fewer papillomas than wildtype controls and did not progress to SCC [57]. Additionally, *Smad3*−/− epidermis and keratinocytes were significantly resistant to the proliferative and proinflammatory effects of TPA, suggesting that Smad3 is critical for tumor promotion by TPA [57]. In contrast to these whole animal knockout studies, when *Smad3*−/− keratinocytes were transduced with a v-*Ras* ^Ha^ oncogene and skin grafted onto athymic mice, they rapidly progressed to SCC, while wildtype controls formed benign papillomas as expected from previous studies [58]. v-*Ras* ^Ha^-transduced *Smad3*−/− keratinocytes were less sensitive to TGF*β*1-induced growth arrest *in vitro* and were able to escape Ras-induced senescence, that is mediated in part through upregulation of TGF*β*1 expression and signaling [72]. Overexpression of Smad3 but not Smad2, accelerated senescence in v-*Ras* ^Ha^-transduced wildtype keratinocytes and rescued the senescence defect in *Smad3*−/− keratinocytes [58]. The ability of TGF*β*1 to induce growth arrest and senescence in v-*Ras* ^Ha^ keratinocytes was linked to the induction of p16ink4a and p19ARF, and this was dependent on intact Smad3 [73]. These results suggest that Smad3 does indeed function as a tumor suppressor in keratinocytes, and these cells are not inherently resistant to malignant conversion. However it is clear that Smad3 function in keratinocytes or other resident or infiltrating cells in the skin are critical for tumor promotion, further studies with epidermal specific deletion of Smad3 will provide insight as to the lack of SCC formation in *Smad3*−/− mice. 

#### 4.2.3. Smad4

In two models of epidermal-specific Smad4 deletion, the mice exhibited progressive hair-loss due to defects in hair follicle cycling, and the majority developed spontaneous development of SCC within 1 year [56, 74]. Tumors were characterized by altered expression of TGF*β*1-regulated cell cycle genes including c-Myc, p21, and p27. Significantly,* Smad4*−/− tumors exhibited inactivation of PTEN and activation of AKT [56], and codeletion of the Smad4 and PTEN resulted in accelerated hair loss and skin tumor formation [74]. Similar results in HNSCC suggest that activation of AKT is a critical event in tumorigenesis mediated by inactivation of the TGF*β*1-signaling pathway.

#### 4.2.4. I-Smads

 Transgenic mice in which Smad7 was targeted to the basal layer of the skin with a keratin 5 promoter exhibited hyperproliferation in the skin and other stratified epithelia, but these animals died within 10 days after birth [75]. More recently, an inducible Smad7 transgenic has been developed, and in these animals, induction of Smad7 during wounding enhanced keratinocyte proliferation and accelerated reepithelialization through effects on keratinocyte migration and stromal cells in the wound [76]. Glick and colleagues used retroviruses to coexpress Smad7 or Smad6 in primary mouse keratinocytes with v-*Ras* ^Ha^ oncogene retroviruses and transplanted these cells onto athymic mice using a skin grafting system [59]. Skin grafts of keratinocytes transduced with v-*Ras* ^Ha^ alone generated papillomas as expected, as did v-*Ras* ^Ha^ and Smad6. In contrast skin grafts of v-*Ras* ^Ha^- and Smad7-transduced keratinocytes rapidly progressed to SCC [59]. These results demonstrate that Smad7 inhibition of TGF*β*1 signaling can drive progression of Ras oncogene expressing primary keratinocytes but BMP signaling and Smad6 inhibition of BMP signaling do not play a significant role in progression in this model. 

### 4.3. Non-Smad-Signaling Pathways

 Many different non-Smad-signaling pathways downstream of the TGF*β* receptor with likely impact on various aspects of the cancer phenotype have been identified using cultured cells [77]. Yet, the importance of this as a component of TGF*β*1 signaling in tumor formation and progression *in vivo* has been more difficult to prove simply because these pathways are activated by many upstream-signaling molecules, and appear to synergize with Smad pathways to generate maximal biological responses [78–80]. The most clearcut evidence for importance of non-Smad signaling by TGF*β* receptors in a cancer phenotype comes from analysis of TGF*β*1 mediated EMT. TGF*β* associated kinase 1 (TAK1) is a MAPK kinase kinase (MAPKKK) family member that is important for TGF*β*-induced activation of the p38 MAPK pathway (Yamaguchi et al. 1995), although it can also activate other pathways such as NFkB and JNK. In NMuMG, mouse mammary epithelial cells knockdown of TRAF6, a key intermediate between T*β*RI and TAK1, blocked the ability of TGF*β*1 to induce EMT, but had no effect on Smad-dependent responses [81]. TGF*β*1 can also induce EMT through activation of the PI3Kinase/Akt/mTOR pathway, and this has been studied in both the murine mammary gland NMuMG cells and human HaCaT keratinocytes [82, 83]. While inhibition of mTORC1 in these cells with rapamycin did not block TGF*β*1-induced EMT [83], inhibition of TGF*β*1-induced activation of mTORC2 did block EMT [84]. Recent reviews provide more detailed analysis of non-Smad signaling pathways and potential impact on cancer [77, 85] and potential targets for inhibition of TGF*β*1 driven invasion and metastasis. However, direct demonstration that these pathways are specifically activated by TGF*βin vivo* is a significant challenge.

### 4.4. TGF*β* Ligand

Although there are three distinct TGF*β* family members, TGF*β*1, *β*2, and *β*3 all of which have been detected in skin and skin tumors, nearly all mouse models have focused on TGF*β*1. Both TGF*β*2 and TGF*β*3 null mice have been generated, and these have distinct developmental defects that lead to perinatal lethality [86, 87]. No skin targeted knockouts of these genes or overexpression models have been developed that would specifically allow determination of a distinct role in carcinogenesis. Increased levels of TGF*β*1 occurs in primary keratinocytes expressing oncogenic v-*Ras* ^Ha^ [88], and TPA and other tumor promoters rapidly induce TGF*β*1 expression in the suprabasal layers of the epidermis [66, 89]. TPA also induces expression of T*β*RII in normal epidermis [90]. Thus TGF*β*1 expression is likely elevated in the microenvironment surrounding an expanding clone of initiated keratinocytes. Overexpression of TGF*β*1 in the epidermis blocks TPA-induced hyperplasia and papilloma formation [90] and *Tgfb1*−/− keratinocytes transduced with a v-Ras^Ha^ retrovirus rapidly form SCC in athymic mouse skin grafts, while *Tgfb1*+/+ keratinocytes develop only benign papillomas [48]. Similarly, benign papillomas with a high risk progression phenotype exhibit reduced expression of TGF*β*1 [31, 32]. In contrast to these studies* Tgfb1*+/− mice develop fewer chemically induced benign tumors than *Tgfb1*+/+ mice, although the tumors formed in *Tgfb1*+/− mice had a higher frequency of malignant conversion [47]. TPA-induced proliferation was reduced in *Tgfb1*+/− skin and in tumors that formed in *Tgfb1*+/− mice. Surprisingly while TPA-induced inflammation was exaggerated in *Tgfb1*+/− skin, tumors formed in *Tgfb1*+/+ mice had increased tumor inflammation, and this was paralleled by elevated proinflammatory cytokine expression in v-*Ras* ^Ha^-transduced *Tgfb1*+/+ keratinocytes compared to *Tgfb1*+/− keratinocytes [47]. These results suggest that within the local microenvironment of the initiated keratinocyte physiological levels of TGF*β*1 function in either an autocrine or paracrine way to enhance tumor outgrowth but act to suppress malignant progression.

 Several transgenic mouse models overexpressing either active or latent TGF*β*1 in the basal layer of the skin exhibit an inflammatory infiltrate coupled with angiogenesis and hyperproliferation [91, 92]. It is possible that elevated TGF*β*1 by itself acts as a tumor promoter, although this has not been directly demonstrated. More likely the effect may be indirect through the actions of inflammatory cytokines produced by infiltrating immune cells which could counteract the growth inhibitory effects of TGF*β*1 on initiated cells [44, 45]. Lesions that develop in mice overexpressing TGF*β*1 have high levels of proinflammatory cytokines and chemokines similar to Th1 inflammatory diseases such as psoriasis [91], and the pattern of gene expression in inflamed skin is similar but not identical to that of psoriasis [93], where TGF*β*1 is also overexpressed in lesional keratinocytes and sera [94, 95]. Expression of TGF*β*1 in the oral mucosa also caused a similar inflammatory and angiogenic response [96]. Thus, in this context, TGF*β*1 overexpression appears to provoke a chronic inflammatory response, although is not yet clear if the inflammatory infiltrate is similar to that following TPA treatment or wounding. Nevertheless, the hyperproliferation is likely due to either downregulation of TGF*β*1-signaling components or secondary factors produced by the inflammatory cells that can stimulate keratinocyte proliferation. The psoriasis-like inflammation that develops in TGF*β*1 overexpressing mice however does not appear dependent on T cells [97] or the IL17/IL23 axis [98]. TGF*β*1 is chemotactic for certain innate immune cells, such as macrophages [99] mast cells [100, 101], and neutrophils [102] and it is possible that directs effects of TGF*β*1 on innate immune cells recruitment to the skin is responsible for the inflammatory phenotype. We have shown recently that as early as 2 days after elevation of TGF*β*1 in the epidermis there is an increased numbers of B220^+^ plasmacytoid dendritic cells (pDCs), Langerin(CD207)^+^ dermal dendritic cells and CD11b^+^ and CD11b^−^ dermal DCs (dDCs) concomitant with increased expression of CD86, a maturation marker in skin-draining lymph nodes (LNs). This was accompanied by increased T cell activation in the LN and an increased contact hypersensitivity responses to topical DNFB. In addition there was a significant influx of plasmacytoid and dermal dendritic cells into the skin following TGF*β*1 induction [103], and pDCs have been strongly linked to the initiation of chronic inflammation in psoriasis [104]. We observed a similar influx of DC into papillomas expressing TGF*β*1, although these were not characterized as completely [46]. Other studies have shown that overexpression of TGF*β*1 in xenotransplanted human SCC lines traps dendritic cells within the tumor [105, 106] thereby allowing escape from antitumor immunity. These results suggest that activation of skin DC by TGF*β*1 is linked to its proinflammatory function in normal skin and this may have significant consequences for the function of this cytokine in skin carcinogenesis.

 In contrast to the suppressive effects of TGF*β*1 overexpression on papilloma formation [45, 90], continuously elevated levels of TGF*β*1 appear to promote formation of highly undifferentiated spindle carcinoma [90], and 15 weeks of TGF*β*1 overexpression in benign papillomas lead to increased invasiveness and metastases [45]. These results support the concept that has been studied *in vitro* in detail that TGF*β*1 can cause an EMT-like phenotype in SCC cells. However it is not clear if the *in vivo* studies represent selection for more malignant cells under the influence of high tissue levels of TGF*β*1, since short-term expression of TGF*β*1 in benign papillomas causes significant tumor regression coupled with a neutrophilic and T cell infiltrate into the tumors [46]. To examine whether TGF*β*1 signaling in tumor cells was required for suppression of EMT and metastasis, Wang and colleagues made compound transgenic mice expressing an inducible TGF*β*1 and delta-T*β*RII transgenes. Here, TGF*β*1 overexpression in late-stage papillomas with wildtype Type II receptor did not inhibit proliferation but increased metastasis and EMT. TGF*β*1-induced EMT was blocked by the delta-T*β*RII transgene, but metastasis was not [30]. Tumors overexpressing TGF*β*1 with blocked TGF*β*1 signaling had greater metastasis than tumors with each transgene alone, although some non-Smad pathways of TGF*β*1 signaling appeared to be intact in the compound transgenic tumors. Thus, it appears that TGF*β*1-mediated EMT is a tumor cell autonomous effect, but metastasis induction may involve changes in the tumor microenvironment or altered TGF*β*1 signaling in tumor cells.

#### 4.4.1. Coreceptors and Binding Proteins

 These proteins regulate interaction of TGF*β*1 with receptors and control extracellular levels of active TGF*β*1 and so are considered here. Although endoglin is expressed primarily on vascular endothelial and smooth muscle cells, it has been detected in normal mouse and human epidermis, in both hair follicles and basal layer of the interfollicular epidermis [107]. Endoglin exists as a membrane bound form but is shed from the membrane at late stages of tumor progression in spindle cell carcinoma [108]. The role of endoglin in skin carcinogenesis was determined using *Eng*+/+ and *Eng*+/− mice [107]. *Eng*+/− mice had significantly reduced numbers of benign papillomas but the tumors that did form were largely SCC and spindle-cell carcinoma. Knockdown of endoglin in transformed keratinocyte cell lines not only enhanced TGF*β*1 signaling, induced growth arrest and suppressed tumor formation, but also caused EMT, invasiveness and conversion to spindle cell carcinoma [108]. Expression of endoglin in a spindle cell carcinoma line suppressed Smad phosphorylation and tumorigenicity [108]. These results suggest that endoglin acts to downmodulate TGF*β*1 signaling in keratinocytes, and generating results similar to the TGF*β*1+/− mice [47], during tumor progression enhances TGF*β*1 signaling, EMT, and progression to spindle-cell carcinoma.

 Activation of latent TGF*β*1 is a complex process that is critical for maintenance of normal tissue homeostasis and rapid release of bioactive TGF*β*1 in response to signals that disrupt the normal tissue microenvironment. LTBP-1 is covalently linked to the propepeptide region of TGF*β*1 and secreted from cells as the large latent complex. To determine the role of LTBP-1 in TGF*β*1 function, Rifkin and colleagues generated mice in which cysteine 33 in both propeptide chains was mutated to serine to prevent disulfide bond formation with LTBP-1 [109]. These animals phenocopied *Tgfb1*−*/*− mice [110], although with a less severe phenotype suggestive of a hypomorphic state due to reduced active TGF*β*1 levels. In addition to the multiorgan inflammation, absence of epidermal Langerhans cells and shortened lifespan, these animals also spontaneously developed stomach, rectal, and anal tumors [109]. While these mice did not develop skin cancers, this model illustrates the critical nature of latent TGF*β*1 activation for generating sufficient TGF*β*1 in the microenvironment for normal tissue homeostasis. 

## 5. Conclusions

The role of TGF*β*1-signaling pathway in the pathogenesis of SCC and other cancers is complex due to the diverse biological processes that are regulated by TGF*β*1 and the cell type and context dependence of specific responses. Nevertheless, sufficient studies have been done to make some general conclusions. First, inactivation or diminution of pathway activity represents a significant component of human SCC pathogenesis, whether by receptor mutation, loss of receptor expression as measured by reduced receptor or pSmad2 levels, or loss of Smad4 expression. However, the mouse models suggest that except for Smad4, inactivation of the TGF*β* pathway by itself is not sufficient for tumorigenesis, despite alterations in tissue homeostasis. It may be that this stems from the centrality of Smad4 in multiple TGF*β*1 superfamily-signaling pathways. Further, the mouse models suggest that Smad2 and Smad3 function in carcinogenesis may be distinct, but this also may depend on what tissue compartment function is inactivated. It remains to be determined whether epidermal specific Smad3 deletion will have similar or distinct effects on cancer development as the Smad2 epidermal null. While the ability of TGF*β*1 pathway inactivation to collaborate with oncogenic Ras has been shown in multiple studies, the finding that PI3-kinase/AKT is activated in tumors from two different models of pathway inactivation, that PTEN deletion cooperates with TGF*β*1 pathway inactivation for tumorigenesis, and that parallel changes occur in human SCC suggests that the interaction of these two pathways is important for SCC pathogenesis and deserves further analysis. It is an accepted paradigm that long-term expression of TGF*β*1 promotes a more malignant phenotype, and this is certainly born out by in vitro studies of TGF*β*1-treated SCC cells and elevated expression of TGF*β*1 in mouse and human cancers where pathway inactivation occurs. Nevertheless, the animal models suggest that increased expression in benign tumors or during the course of cancer induction selects for cells with a more aggressive, metastatic phenotype. The observation that this is enhanced when receptor signaling is blocked suggests that other pathways are activated in the tumor cells or that effects of TGF*β*1 on the tumor microenvironment predominate, where elevated TGF*β*1 leads to significant inflammation. Finally, although nearly all of these studies have been done in the chemical carcinogenesis model, for cutaneous cancer at least, it is not clear if alterations in TGF*β*1 signaling would impact UV-induced skin cancer in the same way. Research on TGF*β* has been one of many surprises. It is certain that many surprises remain in the years ahead. 

## Figures and Tables

**Table 1:** Skin and oral carcinogenesis studies with mouse models of TGF*β*1 signaling.

Signaling component	Mouse model	Study details	Phenotype	Reference
			TGF*β*1 overexpression	
	K6-TGF*β*1*, K10-TGF*β*1 DMBA/TPA	Constitutive and inducible suprabasal expression	Suppressed papilloma formation, increased malignant conversion and spindle cell carcinoma	Cui et al., 1996 [44]
TGF*β*1 ligand	Loricrin-TGF*β*1 gene switch DMBA/TPA	Long-term expression in papillomas	Increased EMT, invasion, and metastasis	Weeks et al., 2001 [45]
	K5rTA x tetOTGF*β*1 DMBA/TPA	Short-term expression in papillomas	Growth arrest, regression, and tumor inflammation	Mohammed et al., 2010 [46]
			TGF*β*1 knockdown	
	*Tgfb1*+/− versus *Tgfb1*+/+ DMBA/TPA	Germline *Tgfb1 *heterozygote	Reduced papillomas in TGF*β*1+/−, increased malignant conversion	Pérez-Lorenzo et al., 2010 [47]
	*Tgfb1*−/−; v-*Ras* ^Ha^ xenotransplantation	Skin grafts of PMEK onto athymic mice	SCC with TGF*β*1−/−, papilloma with TGF*β*1+/− and +/+	Glick et al., 1994 [48]

T*β*RI	DMBA/TPA pharmacological inactivation	Topical SB431542 during TPA promotion	Reduced papilloma, increased conversion	Mordasky Markell et al., 2010 [49]
	DMBA/TPA pharmacological inactivation	Systemic LY2109761 during TPA promotion	Increased malignant phenotype of SCC	Connolly et al., 2011 [50]
	K14Cre^ER^ x *Tgfb*1^fl/fl^ DMBA	deletion of T*β*RI in oral mucosa	Accelerated HNSCC with AKT activation	Bian et al., 2009 [51]
	K14Cre^ER^ x *Tgfb*1^fl/fl^ x *Pten* ^ fl/fl^	deletion of T*β*RI and PTEN in oral mucosa	Accelerated HNSCC	Bian et al., 2012 [52]

T*β*RII	Loricrin-Δ*Tgfbr*2	Epidermal expression of dominant negative type II receptor	Reduced tumor latency, increased SCC	Go et al., 1999 [42] Go et al., 2000 [43]
	K5Cre^Pr1^ x *Tgfbr*2^fl/fl^ DMBA or x K-Ras^12D^	Oral mucosa deletion of T*β*RII	HNSCC only with DMBA or K-Ras	Lu et al., 2006 [53]
	K14-Cre x *Tgfbr*2^fl/fl^	Epidermal deletion of T*β*RII	No skin tumors, spontaneous anogenital SCC	Guasch et al., 2007 [54]
	K14-Cre x *Tgfbr*2^fl/fl^	v-Ras^Ha^ xenotransplantation	Aggressive SCC	Guasch et al., 2007 [54]

R-Smads	K5Cre^Pr1^ x *Smad*2^fl/fl^ DMBA/TPA	Basal/stem cell deletion of Smad2 in epidermis	Increased tumors accelerated more aggressive SCC	Hoot et al., 2008 [38] Hoot et al., 2010 [55]
	MMTV-Cre x *Smad*4^fl/fl^	Epidermal deletion of Smad4	Hair follicle defects spontaneous SCC	Qiao et al., 2006 [56]
	K5Cre^Pr1^ x *Smad*4^fl/fl^	Deletion of Smad4 in oral mucosa	Spontaneous HNSCC w/genomic instability increased inflammation normal and tumor tissue	Bornstein et al., 2009 [35]
	Smad3−/− DMBA/TPA	germline Smad3 null	Suppressed tumor formation, resistance to TPA	Li et al., 2004 [57]
	*Smad3*−*/*−; v-Ras^Ha^	Primary mouse keratinocyte skin grafts	Progression to SCC	Vijaychandra et al., [58]

I-Smads	Smad7 + v-Ras^Ha^ Smad6 + v-Ras^Ha^	Primary mouse keratinocyte skin grafts	Smad7: rapid progression to SCC Smad6: papilloma	Liu et al., 2003 [59]

TGF*β*1/T*β*RII	TGF*β*1 gene switch x Δ*Tgfbr*2 DMBA/TPA	Inducible expression of TGF*β*1 in papillomas with inhibition of TGF*β* receptor	Suppressed EMT in papillomas, increased metastasis	Han et al., 2005 [30]

*Unless otherwise indicated TGF*β*1 transgene used was TGF*β*1^S223/S225^ constitutively active mutant

fl/fl: floxed alleles.

Δ: truncation of cytoplasmic domain generating dominant negative receptor.

DMBA/TPA indicates 2-stage chemical carcinogenesis protocol.

Cre^ER^: tamoxifen-inducible Cre recombinase.

Cre^Pr1^: rU486 inducible Cre recombinase.

## Abbreviations

DMBA: 7,12-Dimethylbenz(a)anthracene
EMT: Epithelial-to-mesenchymal transition
HNSCC: Human head-&-neck squamous cell carcinoma
IHC: Immunohistochemistry
SCC: Squamous cell carcinoma
*Tgfbr-1*: Type I TGF-*β* receptor gene
*Tgfbr-2*: Type II TGF-*β* receptor gene
*Tgfb1*: Murine transforming growth factor beta gene
TGF*β*: Transforming growth factor-*β*
TPA: 12-tetradecanoyl-phorbol-13-acetate
T*β*RI: Type I TGF-*β* receptor
T*β*RII: Type II TGF-*β* receptor.
